# Correlation Between Thyroid Nodules and Metabolic Syndrome: A Systematic Review and Meta-Analysis

**DOI:** 10.3389/fendo.2021.730279

**Published:** 2021-09-16

**Authors:** Chenyu Zhang, Xiaotong Gao, Yutong Han, Weiping Teng, Zhongyan Shan

**Affiliations:** Department of Endocrinology and Metabolism, NHC Key Laboratory of Diagnosis and Treatment of Thyroid Diseases, Institute of Endocrinology, The First Affiliated Hospital of China Medical University, Shenyang, China

**Keywords:** thyroid nodules, metabolic syndrome, meta-analysis, thyroid, iodine-deficient

## Abstract

**Objective:**

Thyroid nodules (TNs) are a common thyroid disorder that can be caused by many factors. Several studies have investigated the relationship between TNs and metabolic syndrome (MetS), but the role of sex and age remains controversial. The purpose of this paper was to analyze published data from all relevant studies to reliably estimate the relationship between TNs and MetS.

**Methods:**

Thirteen articles were included in this study; articles were identified by searching for publications until July 2021 in PubMed, EMBASE, the Cochrane Library and the Web of Science. The outcomes are presented as the summary odds ratio (OR) and 95% confidence interval (CI) and the pooled prevalence and 95% CI.

**Results:**

The TNs prevalence was significantly higher in MetS patients than in controls (OR 1.88, 95% CI 1.42-2.50, P < 0.0001) and was independent of sex (male: OR 1.53, 95% CI 1.20-1.94, P = 0.0006; female: OR 1.90, 95% CI 1.54-2.33, P < 0.00001; combined: OR 2.06, 95% CI 1.31-3.25, P = 0.002) and age (< 40 years old: OR 1.62, 95% CI 1.39-1.89, P < 0.0001; 40~50 years old: OR 2.14, 95% CI 1.49-3.08, P < 0.0001;50~60 years old: OR 1.50, 95% CI 1.08-2.07, P = 0. 01; 60 years old: OR 1.70, 95% CI 1.36-2.14, P < 0.00001); the pooled TNs prevalence in MetS patients was 45% (95% CI 36-54%). However, it has not yet been considered that MetS is related to TNs in people with iodine deficiency (OR 3.14, 95% CI 0.92-10.73, P = 0.07).

**Conclusion:**

The meta-analysis results showed a strong correlation between TNs and MetS. Both male and female patients with MetS had an increased TNs prevalence. In addition, the prevalence was independent of age. However, MetS is not considered to be associated with TNs in iodine-deficient populations.

## Introduction

Thyroid nodules (TNs) constitute one of the most common clinical thyroid disorders. A recent nationwide cross-sectional survey indicated that the prevalence of TNs in mainland China is as high as 20.43% (1). Approximately 5% of patients have palpable nodules, and up to 70% of patients have nodules that are found incidentally on ultrasonography (2), indicating the high prevalence of occult nodules. In addition, the prevalence of TNs is higher in women, elderly individuals, iodine-deficient individuals and those with radiation exposure than in their counterparts (3–5). Approximately 5%-10% of thyroid nodules are malignant (6, 7). The high prevalence of thyroid nodules may mean an increased possibility of thyroid cancer. Therefore, risk factors associated with TNs have received significant attention in recent years.

A variety of metabolic diseases are considered to be important risk factors for TNs (8–10), but this finding needs to be further validated. Some cross-sectional studies have shown a close association between thyroid disease and metabolic disorders (i.e., metabolic syndrome [MetS] and its components) (11–13). MetS is characterized by a cluster of abnormal metabolic parameters consisting of insulin resistance, central obesity, type 2 diabetes, impaired glucose tolerance, hyperinsulinemia, and dyslipidemia (14). The global prevalence of MetS is between 11.6% and 62.5% (15). One study indicated that the prevalence of MetS in mainland China was 28.6% (11). Studies have found that obesity and insulin resistance are strongly correlated with TNs (10, 16). However, the overall relationship between MetS and TNs is controversial, and whether the relationship is influenced by sex- or age- is still unclear. Some studies found that TNs were significantly related to an increased risk of MetS, and males and elderly people (≥60 years old) with MetS had a higher risk of TNs than females and younger people (<60 years old) (9). However, another study found that in the MetS population over 45 years old, the prevalence of TNs in women was one-third higher than that in men (38.5% versus 26%) (17). In addition, after adjustment for age, smoking and alcohol consumption, MetS was an independent risk factor for TNs in females but not in males (17). A relevant meta-analysis is currently needed to summarize and clarify the potential associations, especially among subgroups with different demographic characteristics.

This study is a comprehensive systematic review and meta-analysis of all published case-control and cross-sectional studies that evaluated the correlation between MetS and TNs and conducted further investigation in sub-analyses according to sex, age, iodine nutrition status, study type and diagnostic criteria.

## Methods and Materials

### Search Strategy

**Figure 1:**
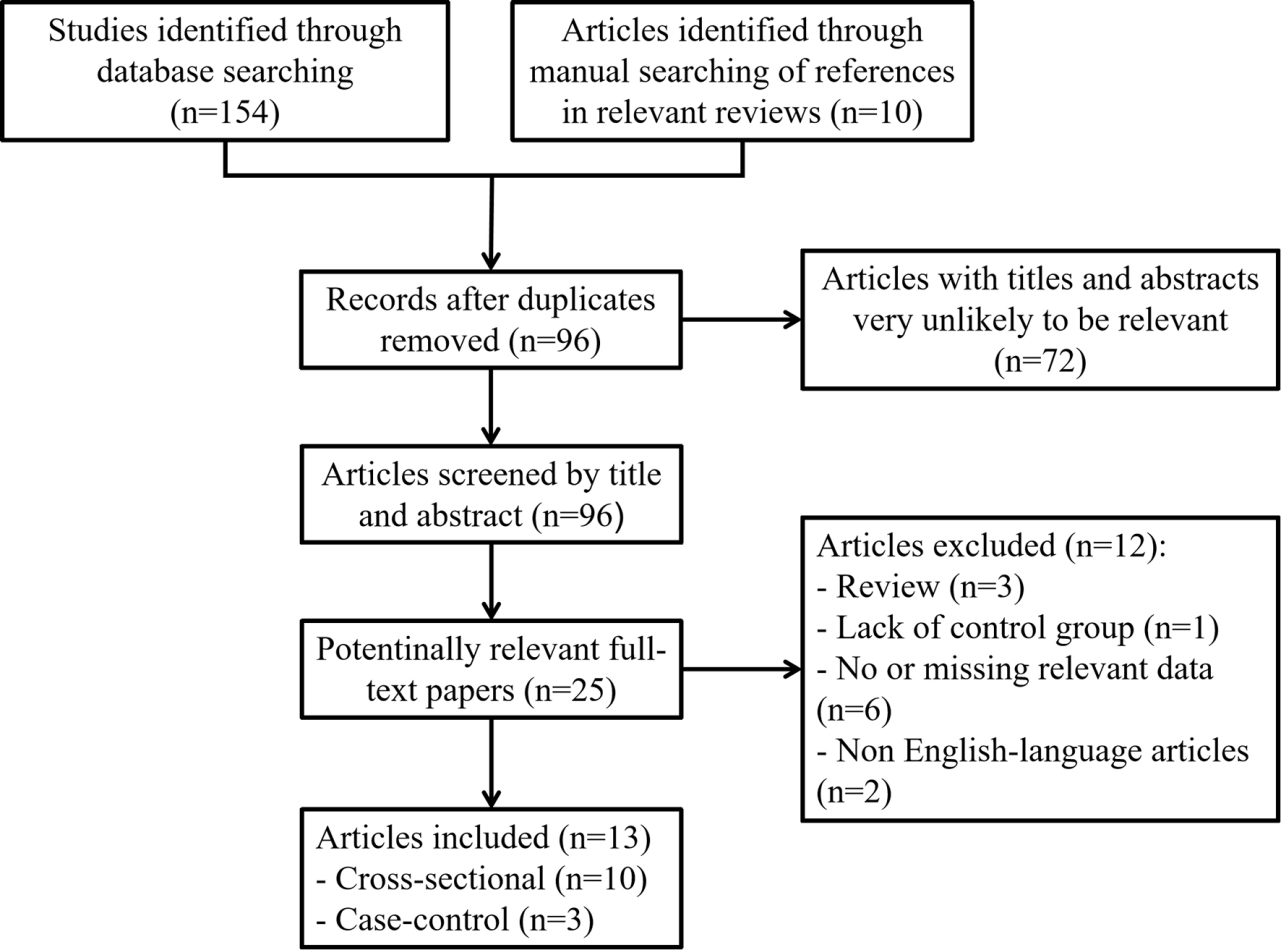
Flow chart showing the detailed inclusion or exclusion procedure. Thirteen independent articles were included in the meta-analysis.

The criteria for conducting and reporting a meta-analysis of observational studies have been reported in extensive detail elsewhere (18). Two researchers (CZ and XG) independently searched for articles published until July 2021 in the PubMed (http://www.ncbi.nlm.nih.gov/pubmed), Web of Science (http://apps.webofknowledge.com), Cochrane Library (http://www.thecochranelibrary.com) and EMBASE (http://www.embase.com) databases. The search terms were as follows: (‘Nodule, Thyroid’ OR ‘Nodules, Thyroid’ OR ‘Thyroid Nodules’) AND (‘Metabolic Syndromes’ OR ‘Syndrome, Metabolic’ OR ‘Syndromes, Metabolic’). We limited the included studies to those published in English. The search and inclusion criteria were restricted to observational research. We also searched for and screened the full texts of references from relevant original papers to identify potentially eligible publications. The selection process is shown in **Figure 1**. The retrieved results in.nbib and.ris formats were exported from the databases and collated in a specific library in the reference manager software EndNote^®^ version X9 (Thomson Reuters, New York, USA).

### Selection Criteria

We used EndNote^®^ version X9 to exclude duplicate articles. When the title or summary information was not enough to judge eligibility, we read the full text. The study inclusion criteria were as follows: (1) the study design was observational, and the publication language was English; (2) all persons included in the exposure group were MetS patients who had undergone clinical evaluation for MetS; (3) all persons included in the control group were without MetS; (4) the outcomes of interest were TN prevalence in patients with MetS; and (5) sufficient information to calculate the combined prevalence or complete subgroup analysis based on factors such as sex, age and iodine nutrition status. When outcomes in the same population were reported in multiple articles, the most detailed report was included. In contrast, studies were immediately excluded if they met one of the following criteria: (1) the article was a case report, letter, review, editorial, or expert opinion; (2) missing necessary information; (3) missing necessary data to calculate prevalence; or (4) no explicit mention of MetS or diagnostic criteria for TNs.

### Data Extraction

The following data were extracted by authors (CZ and XG): first, basic information (first author name, year of publication, location and research design); second, participant characteristics (sample size, number of cases and controls, age, sex, and iodine nutrition status); and third, MetS and TN diagnostic standards. In observational studies, the mean age and body mass index (BMI) in the different sexes were annotated (if any). Two examiners organized the extracted data through Microsoft Excel^®^ spreadsheets, cross-checked the data and independently assessed the quality of each study based on the Newcastle-Ottawa Quality Assessment Scale (19) and the Agency for Healthcare Research and Quality (AHRQ) quality assessment criteria. According to the score for each study, it was classified as low (<4), medium (4-7) or high quality (>8). Any existing differences were discussed and resolved by the two authors until they finally reached a consensus. After assessment, all included studies were found to be of moderate or high quality (shown in **Tables S1** and **S2**).

### Statistical Analyses

We used Review Manager version 5.2 software to calculate odds ratios (ORs) and the corresponding 95% confidence intervals (95% CIs) to assess the strength of the association between TNs and the risk of MetS. We used the pooled prevalence and 95% CIs to assess the prevalence of TNs in MetS patients. The significance of the combined OR was determined by the Z test using the Mantel-Haenszel method, and a P value < 0.05 was considered significant. We used the Cochran Q test (P < 0.05 indicated statistical significance) and the I^2^ statistic to assess heterogeneity. I^2^ values of 25, 50, and 75% indicated low, moderate, and high heterogeneity, respectively. When a high degree of heterogeneity was found, a random-effects model was used to pool the results; if heterogeneity was low, a fixed-effect model was used to pool the results.

To assess the stability of the results, we performed a sensitivity analysis by omitting one report at a time and recomputing the pooled estimates of the remaining studies. Funnel plots were constructed to assess publication bias.

## Results

### Characteristics of the Included Studies

First, we searched the PubMed, EMBASE, Cochrane Library and Web of Science databases and searched for references in relevant reviews and found a total of 164 potentially relevant studies. In total, 96 items remained after removing duplicate records. By carefully screening the titles and abstracts, we selected 25 articles for further assessment. After reading the full texts and applying the inclusion and exclusion criteria, a total of 13 original studies were eventually included in the systematic review and meta-analysis (**Figure 1**). There were two types of studies, including Ten cross-sectional studies and three case-control studies. The basic characteristics of each study included in this meta-analysis are summarized in **Table 1**.

**Table 1:** Characteristics of the studies included in this review.

Author	Year	Country	ST	Iodine nutrition status	Sample size *n total*	Mean age	MetS (+)		MetS (-)		MetS criteria
							N	M/F	N	M/F	
1. Ayturk (20)	2009	Turkey	CC	Iodine-deficient	539	42.7 ± 13.6	278	92/186	261	80/181	NCEP-ATPIII
2. Chen (21)	2018	China	CS	Iodine-adequate	9898	53.34 ± 13.07	2421	852/1569	7477	3265/4212	IDF
3. Ding (17)	2017	Chins	CS	Iodine-adequate	6365	58.8 ± 7.2	2481	965/1516	3884	2105/1779	IDF
4. Feng (22)	2016	China	CS	Iodine-adequate	6494	49.7 ± 3.1	1394	575/819	5100	1852/3248	IDF
5. Guo (9)	2019	China	CS	Iodine-adequate	2606	43.08 ± 15.51	767	463/304	1839	875/964	NCEP-ATPIII
6. Li (1)	2019	China	CS	Iodine-adequate	2068	41.8 ± 13.7	120	99/21	1948	700/1248	CDS
7. Liang (23)	2020	China	CC	Iodine-adequate	4749	37.54 ± 10.07	865	576/289	3884	1949/1935	NCEP-ATPIII
8. Moon (24)	2018	Korea	CS	Iodine-adequate	63259	49.5 ± 10.3	13638	–	48621	–	AHA
9. Pan (25)	2020	China	CS	Iodine-adequate	2040	43.9 ± 11.8	374	–	1666	–	CDS
10. Rendina (26)	2012	Italy	CS	Iodine-deficient	1422	64.2 ± 3.2	461	–	961	–	AHA
11. Shin (27)	2016	Korea	CC	Iodine-adequate	1990	49.8 ± 10.0	253	109/144	1737	582/1155	NCEP-ATPIII
12. Su (28)	2019	China	CS	Iodine-adequate	927	45.7 ± 12.7	437	205/232	490	160/330	IDF
13. Yin (29)	2014	China	CS	Iodine-adequate	13522	Not stated	2774	2234/540	10748	6692/4056	IDF

ST, study type; CC, case-control study; CS, cross-sectional study; N, number of samples; M, male; F, female; NCEP-ATP III, National Cholesterol Education Program Adult Treatment Panel III; IDF, International Diabetes Federation; CDS, China Diabetes Society; AHA, American Heart Association.

### Meta-Analysis Results

**Figure 2:**
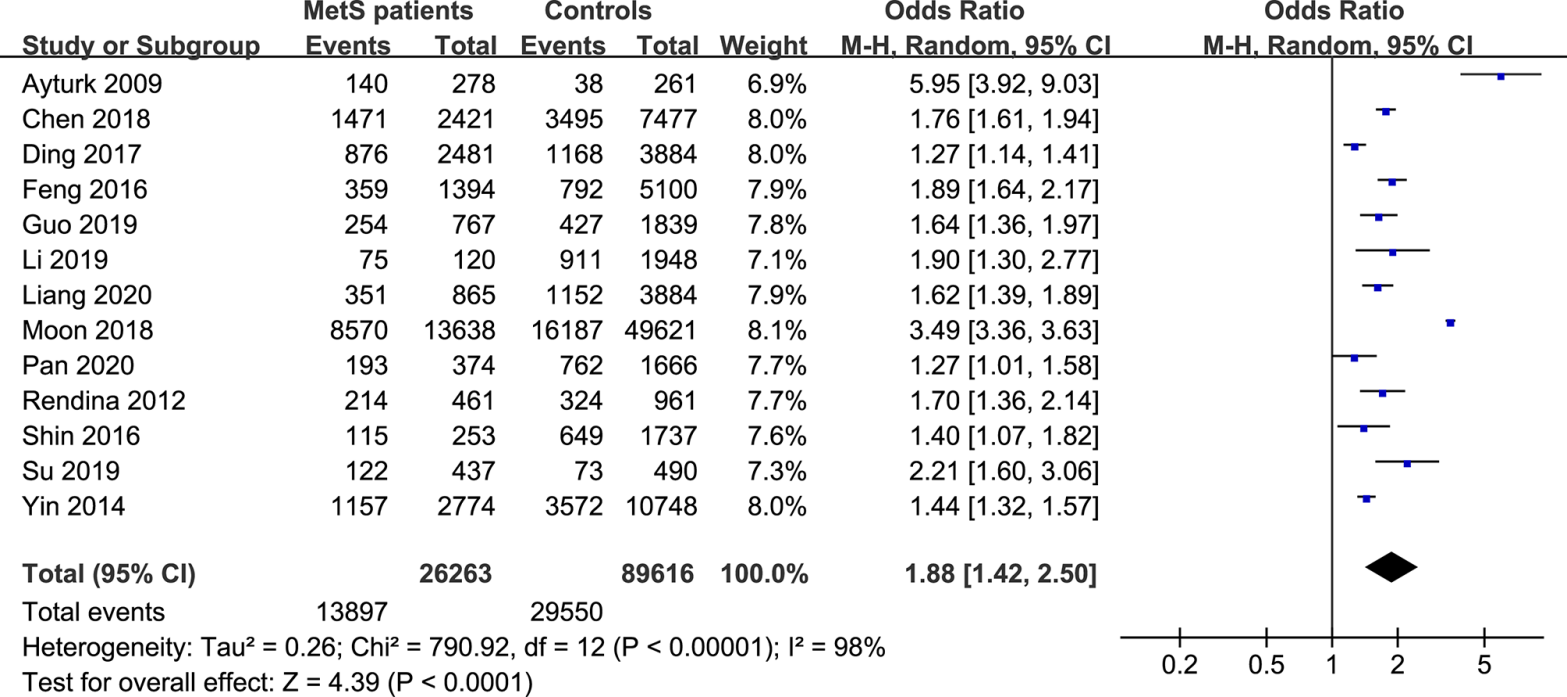
Forest plot of the prevalence of TNs in MetS patients and non-MetS patients (controls).

**Figure 3:**
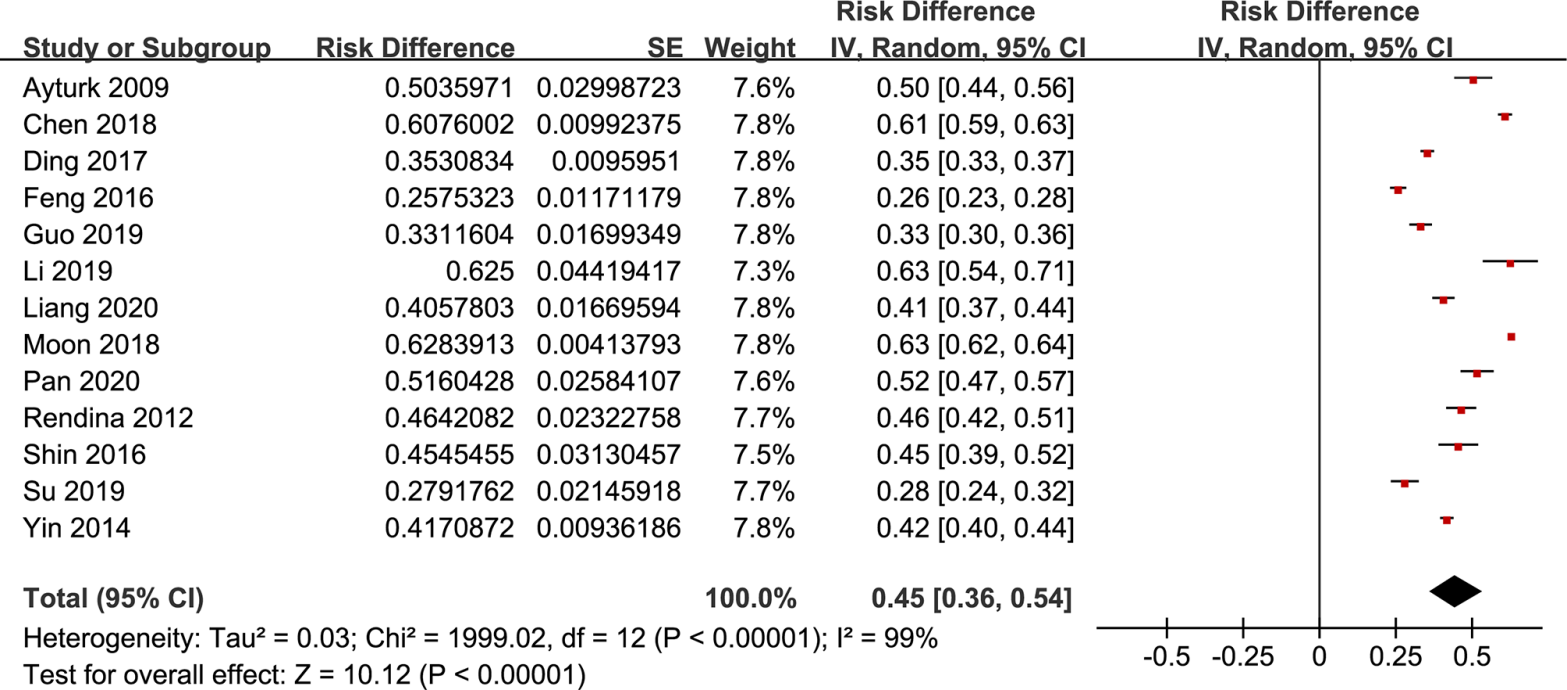
Prevalence of TNs in MetS patients.

**Figure 4:**
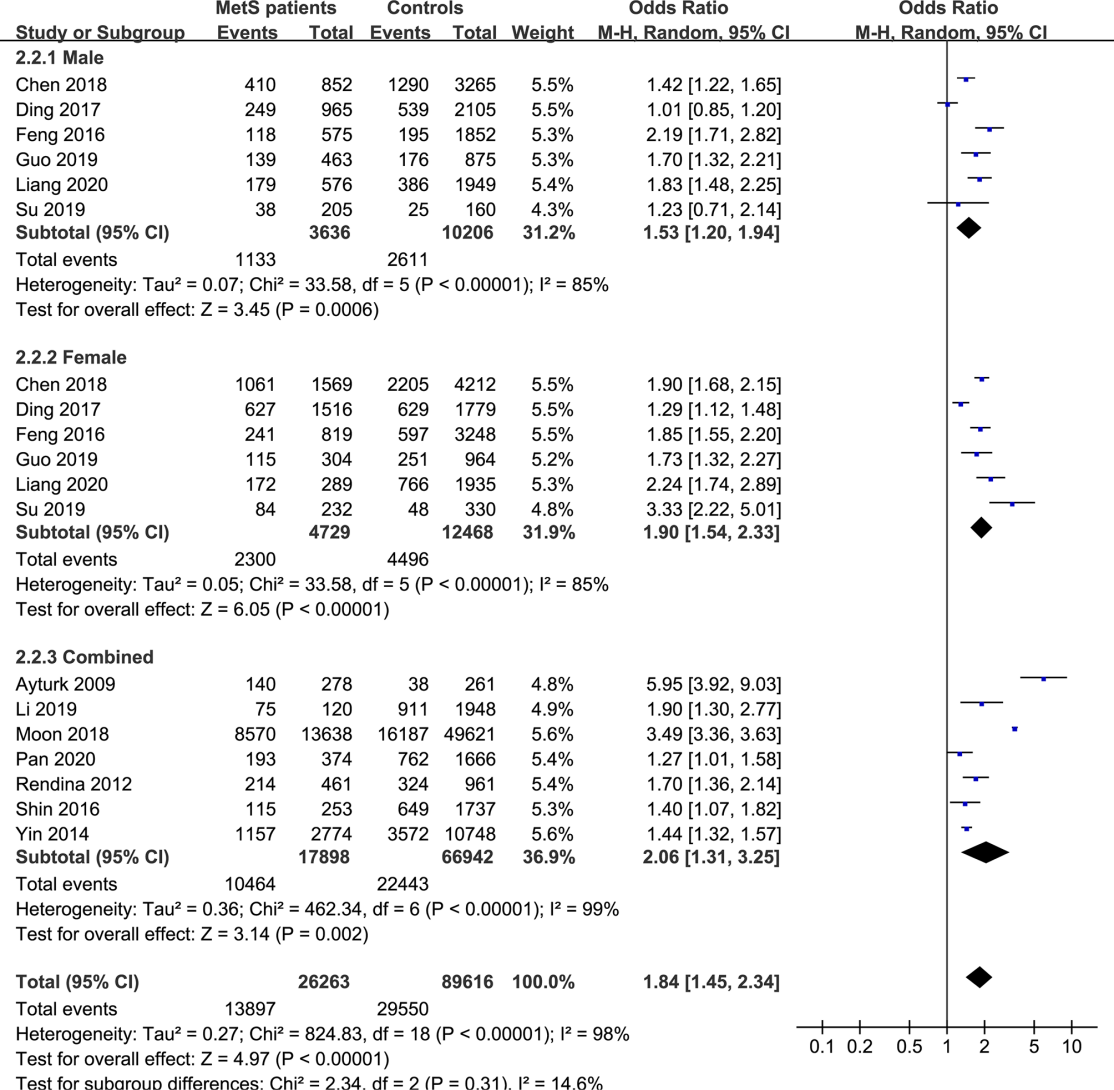
Pooled effect size forest plots of TNs prevalence in MetS patients *versus* non-MetS patients according to sex (male, female and combined groups).

**Figure 5:**
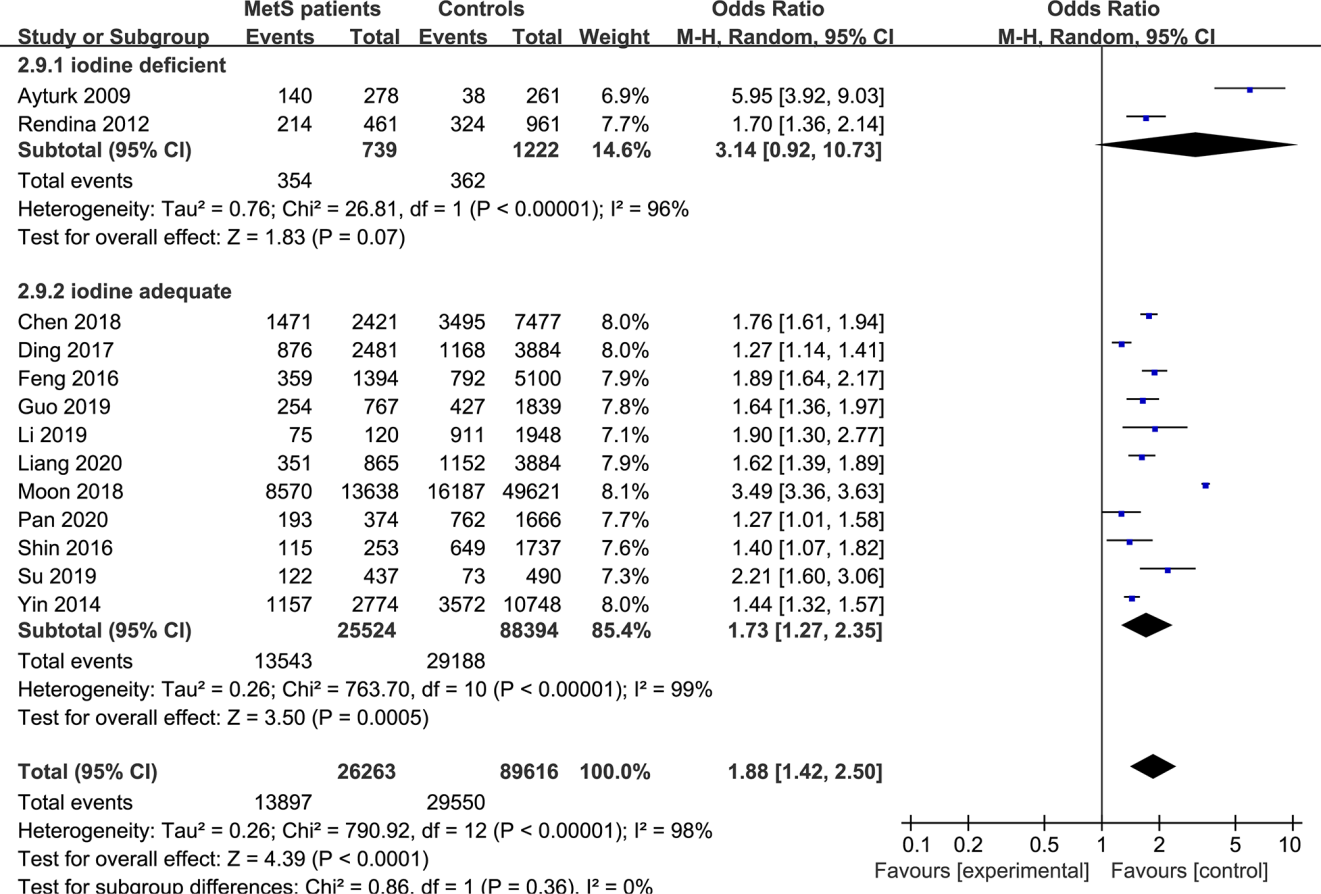
Pooled effect size forest plots of TNs prevalence in MetS patients versus non-MetS patients according to iodine nutrition status (iodine-deficient and iodine-adequate).

The forest plots of the meta-analysis of TNs and MetS are provided in **Figures 2**–**5**. The results of the meta-analysis of the prevalence of TNs in MetS patients versus non-MetS patients (OR 1.88, 95% CI 1.42-2.50, P < 0.0001, I^2^ = 98%) showed that patients with MetS were more likely to have TNs than controls (**Figure 2**). The pooled prevalence of TN in MetS patients was 45% (95% CI 36-54%) (**Figure 3**).

**Figure 6:**
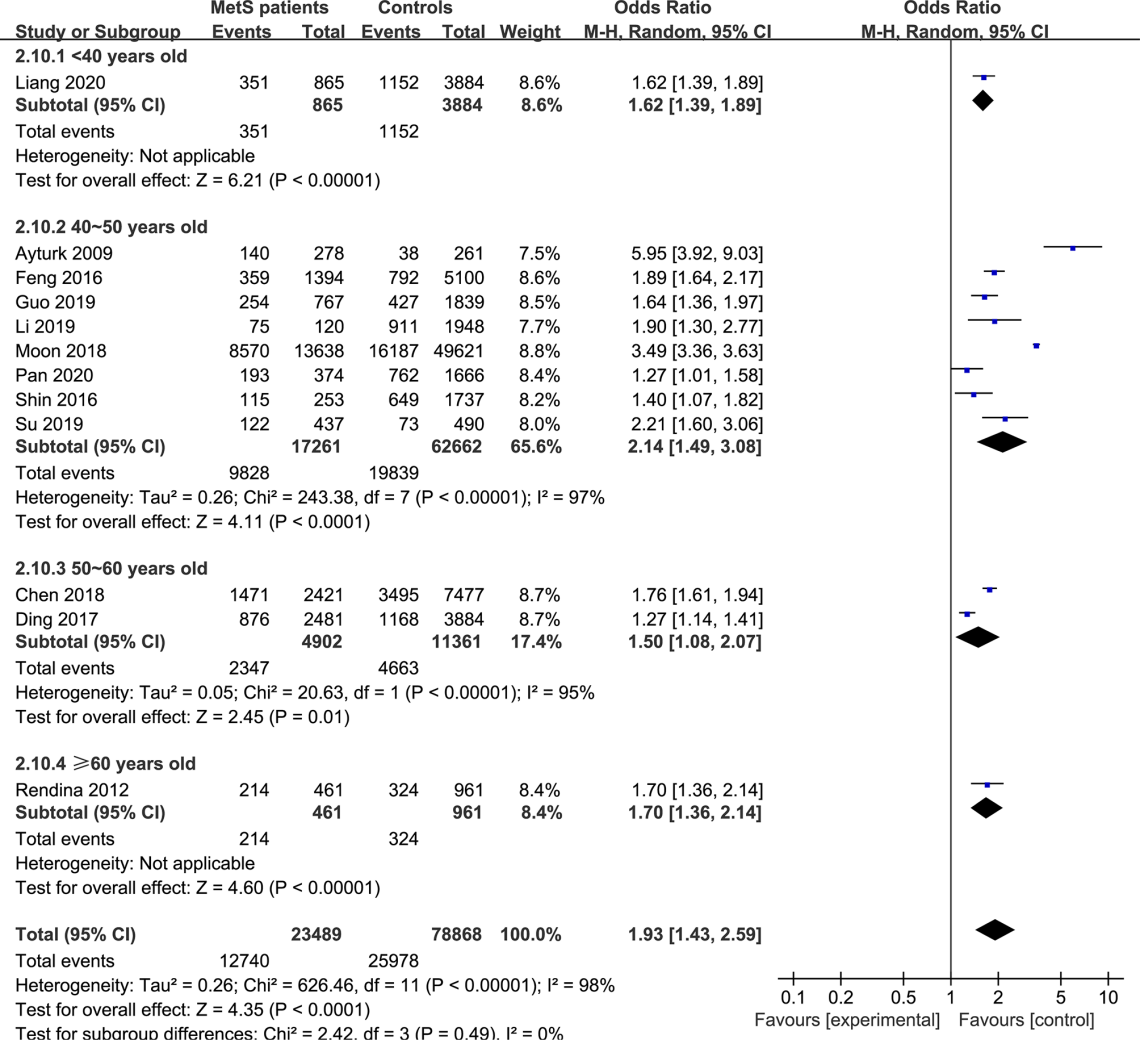
Pooled effect size forest plots of TNs prevalence in MetS patients versus non-MetS patients according to mean age (< 40 years old, 40 ~ 50 years old, 50 ~ 60 years old and ≥60 years old groups).

In the subgroup analysis of the prevalence of TNs in MetS patients, the study population was divided into male, female and combined groups. The results showed that the prevalence of TNs was significantly higher in MetS patients than in non-MetS patients (male: OR 1.53, 95% CI 1.20-1.94, P = 0.0006, I^2^ = 85%; female: OR 1.90, 95% CI 1.54-2.33, P < 0.00001, I^2^ = 85%; combined: OR 2.06, 95% CI 1.31-3.25, P = 0.002, I^2^ = 99%) (**Figure 4**). In addition, we divided the studies into iodine-deficient groups and iodine-adequate groups according to the iodine nutrition status. The results showed that there was a positive relationship between TNs and MetS in the iodine-adequate group (OR 1.73, 95% CI 1.27-2.35, P = 0.0005, I^2^ = 99%); however, we cannot yet suggest a correlation between TNs and MetS in the iodine-deficient population (OR 3.14, 95% CI 0.92-10.73, P = 0.07, I^2^ = 96%) (**Figure 5**). We divided the studies into < 40 years old, 40 ~ 50 years old, 50 ~ 60 years old and ≥60 years old subgroups according to the mean age. The results of this subgroup analysis showed positive associations between TNs and MetS in the < 40 years old (OR 1.62, 95% CI 1,39-1.89, P < 0.00001), 40 ~ 50 years old (OR 2.14, 95% CI 1.49-3.08, P < 0.00001, I^2^ = 97%), 50 ~ 60 years old (OR 1.50, 95% CI 1.08-2.07, P < 0.00001, I^2^ = 95%) and ≥60 years old (OR 1.70, 95% CI 1.36-2.14, P < 0.00001) subgroups (**Figure 6**).

Then, we divided the studies into cross-sectional (CS) and case-control (CC) study subgroups according to the type of study. The results indicated that there was a positive relationship between TNs and MetS in both the CS (OR 1.78, 95% CI 1.28-2.46, P =0.0005, I^2^ = 99%) and CC (OR 2.31, 95% CI 1.21-4.42, P =0.01, I^2^ = 95%) groups. We also divided the studies into National Cholesterol Education Program’s Adult Treatment Panel III (NCEP-ATPIII) (30), International Diabetes Federation (IDF) (31), China Diabetes Society (CDS) (32) and American Heart Association (AHA) (33) subgroups according to the diagnostic criteria for MetS (**Table 2**). The subgroup analysis results showed that among these subgroups, the prevalence of TNs was higher in the MetS group than in the non-MetS group (NCEP-ATPIII: OR 2.07, 95% CI 1.39-3.09, P = 0.0004, I^2^ = 92%; IDF: OR 1.63, 95% CI 1.39-1.92, P < 0.00001, I^2^ = 89%; CDS: OR 1.50, 95% CI 1.01-2.23, P = 0.04, I^2^ = 92%; AHA: OR 2.46, 95% CI 1.22-4.97, P = 0.01, I^2^ = 97%) (**Table 3**).

**Table 2:** Different MetS Diagnosis Criterion.

MetS Diagnosis Criterion	
NCEP-ATPIII (30)	(1) abdominal obesity, defined as a waist circumference (WC) in men >102 cm and in women 88 cm; (2) serum triglycerides (TGs) 150 mg/dl or greater; (3) serum high-density lipoprotein (HDL) in men < 40 mg/dl and in women < 40 mg/dl; (4) blood pressure 130/85 mmHg; (5) fasting plasma glucose 110 mg/dl.
IDF (31)	central obesity (defined as a WC ≥ 90 cm and ≥ 80 cm for Chinese men and women, respectively, with other values for other ethnicities) plus any two of the following four factors: (1) raised TG level (≥1.7 mM, 150 mg/dL) or specific treatment for this lipid abnormality; (2) reduced high-density lipoprotein cholesterol (HDL-c) (<1.03 mM, 40 mg/dL in men and <1.29 mM, 50 mg/dL in women) or specific treatment for these lipid abnormalities; (3) raised blood pressure (BP) (systolic ≥ 130 mmHg or diastolic ≥ 85 mmHg) or treatment of previously diagnosed hypertension; (4) raised fasting plasma glucose (FPG ≥ 5.6 mM, 100 mg/dL) or previously diagnosed type 2 diabetes.
CDS (32)	≥3 components, which are listed as follows: (1) overweight or obesity: BMI ≥ 25 kg/m^2^; (2) dyslipidemia: triglycerides ≥1.7 mmol/L and/or fasting HDL cholesterol <0.9 mmol/L in male or <1.0 mmol/L in female; (3) hypertension: blood pressure ≥140/90 mmHg and/or medication; (4) glucose intolerance: fasting plasma glucose ≥6.1 mmol/L and/or medication.
AHA (33)	at least three of the following five conditions: (1) fasting glucose ≥ 100 mg/dL or receiving drug therapy for hyperglycemia; (2) blood pressure ≥ 130/85 mmHg or receiving drug therapy for hypertension; (3) triglycerides ≥ 150 mg/dL or receiving drug therapy for hypertriglyceridemia; (4) HDL-C < 40 mg/dL in men or < 50 mg/dL in women or receiving drug therapy for reduced HDL-C; and (5) waist circumference ≥ 90 cm in men or ≥ 80 cm in women.

**Table 3:** Meta-analysis of subgroups.

Type of subgroup	Eligible studies	OR (95% CI)	P value	Heterogeneity test	Effect model
**Sex**					
Male	6	1.53 (1.20, 1.94)	=0.0006	p < 0.00001, I^2^ = 85%	Random
Female	6	1.90 (1.54, 2.33)	<0.00001	P < 0.00001, I^2^ = 85%	Random
Both	7	2.06 (1.31, 3.25)	=0.002	P < 0.00001, I^2^ = 99%	Random
**Age**					
< 40 years old	1	1.62 (1.39,1.89)	<0.00001	Not applicable	Random
40 ~ 50 years old	8	2.14 (1.49, 3.08)	<0.0001	P < 0.00001, I^2^ = 97%	Random
50 ~ 60 years old	2	1.50 (1.08, 2.07)	=0.01	P < 0.00001, I^2^ = 95%	Random
60 years old	1	1.70 (1.36, 2.14)	<0.00001	Not applicable	Random
**Iodine nutrition status**					
Iodine-deficient	2	3.14(0.92, 10.73)	=0.07	P < 0.00001, I^2^ = 96%	Random
Iodine-adequate	11	1.73(1.27, 2.35)	=0.0005	P < 0.00001, I^2^ = 99%	Random
**Study type**					
CS	10	1.78 (1.28, 2.46)	=0.0005	P < 0.00001, I^2^ = 99%	Random
CC	3	2.31 (1.21,4.42)	=0.01	P = 0.01, I^2^ = 95%	Random
**Criteria**					
IDF	5	1.63 (1.39,1.92)	<0.00001	P < 0.00001, I^2^ = 89%	Random
CDS	2	1.50 (1.01, 2.23)	=0.04	P = 0.07, I^2 =^ 69%	Random
NCEP-ATPIII	4	2.07 (1.39, 3.09)	=0.0004	P < 0.00001, I^2^ = 92%	Random
AHA	2	2.46 (1.22, 4.97)	=0.01	P < 0.00001, I^2^ = 97%	Random

CS, cross-sectional study; CC, case-control study; N, number of samples; M, male; F, female; NCEP-ATP III, National Cholesterol Education Program Adult Treatment Panel III; IDF, International Diabetes Federation; CDS, China Diabetes Society; AHA, American Heart Association.

### Sensitivity Analysis and Publication Bias

We next performed sensitivity analyses to determine whether modifying the inclusion criteria for the systematic review and meta-analysis affected the final results. Each study included in this systematic review and meta-analysis was removed to reflect the effect of the individual study on the pooled OR. The corresponding pooled ORs did not change significantly, indicating that our results were stable and plausible.

Heterogeneity was inevitable due to the large number of cross-sectional studies included. Hence, we used a random-effects model to complete the analysis. After conducting multiple subgroup analyses, we found that the correlation between TN and MetS could not yet be considered in the iodine deficiency subgroup. Therefore, differences in iodine nutritional status may explain some of the heterogeneity.

**Figure 7:**
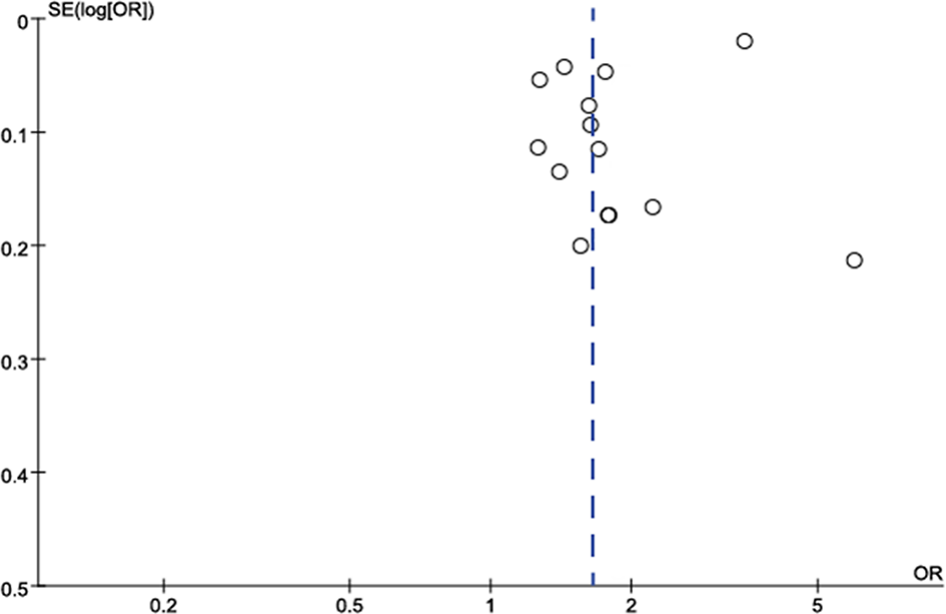
Funnel plot to assess publication bias in studies on the association between TNs and the risk of MetS. Each point represents a separate study for the indicated association. Log OR, natural logarithm of OR. The vertical line represents the effect size.

The publication bias in the meta-analysis of the eight included studies is shown in **Figure 7**, with funnel plots showing publication bias in two small samples of studies.

## Discussion

Our meta-analysis quantitatively assessed the association of TNs with MetS by including and evaluating thirteen independent observational studies. We reported that MetS was related to a 1.88-fold increase in the risk of TNs (95% CI 1.42-2.50). The pooled TNs prevalence in MetS patients was 45% (95% CI 36-54%). To our knowledge, this is the first meta-analysis to examine the relationship between TNs and MetS to date.

MetS is a fairly common endocrine syndrome. Since the establishment of the definition by the NCEP-ATPIII in May 2001, it has received much attention, with 81,254 relevant publications in PubMed to date and the establishment of various diagnostic criteria, such as the NCEP-ATPIII, IDF, CDS and AHA criteria. One important question in MetS research is whether MetS is associated with other diseases (34), for example, whether MetS is predictive of coronary heart disease (35). A large cross-sectional study from China, the Thyroid Disorders, Iodine Status and Diabetes (TIDE): a National Epidemiological Survey Project, investigated the prevalence of metabolic and thyroid diseases in 31 Chinese provinces and cities and found a strong correlation between MetS or its components and thyroid dysfunction, thyroid antibodies and urinary iodine concentration (11–13, 16).

TNs is one of the most common thyroid disorders. However, most of the TN patients diagnosed in our clinical practice are asymptomatic and there is no theoretical basis regarding whether TNs have a potential impact on the metabolic index. But we found that there was a strong correlation between TNs and MetS. Therefore, we speculated that metabolic disorders may be a risk factor for thyroid nodules. Some studies have shown that patients with more MetS components are more likely to develop thyroid nodules than patients without or with one MetS component (27). In addition, patients with poorly controlled MetS, especially those with uncontrolled abnormal glucose metabolism, are at higher risk of developing thyroid nodules (27). Studies have shown that insulin resistance is positively correlated with the prevalence of thyroid nodules (10). Insulin resistance may directly activate the proliferation pathway through insulin or insulin-like growth factor 1, regulating the expression of thyroid genes and the proliferation and differentiation of thyroid cells (36). In addition, insulin resistance may also be related to the thyroid vascular structure (37), thereby promoting the formation of thyroid nodules. Whether abnormalities in other metabolic indicators can promote the formation of thyroid nodules through certain channels still needs further research.

Moreover, it is still unclear whether the abovementioned hypothesis will be maintained in subgroups with different demographic characteristics. The two previous studies have reached opposite conclusions at different levels of gender and age (9, 17). Subgroup analyses were used to evaluate the effects of sex and age characteristics on this association. The results of the meta-analysis showed that when MetS patients were analyzed according to sex, the prevalence rates of TNs were increased in men, women and combined groups, and the differences were not significant, indicating that the increased prevalence of MetS in TN patients was independent of sex. The differences were also not significant in the subgroup analysis according to age, so it cannot be assumed that the prevalence of MetS differs among TN patients < 40 years old, 40 ~ 50 years old, 50 ~ 60 years old and ≥60 years old. However, we cannot yet suggest an association between TNs and MetS in the iodine-deficient population. As is known to all, iodine plays a key role in the occurrence and development of thyroid diseases. Too high or too low of iodine consumed by the human body may actually cause the appearance of TNs (38). Multiple thyroid nodules are more common in the case of iodine excess, while single nodules are more common in the case of iodine deficiency (39). In addition to TN, iodine nutrition is also related to the occurrence of MetS. One cross-sectional studies demonstrate a correlation between the urinary iodine concentration (UIC) and metabolic-related diseases which presented as a U-shaped curve (11) and iodine deficiency was a risk factor for hypertension and hypercholesterolemia. The physiological function of iodine in the human body mainly depends on its characteristics as a halogen element. Iodine is highly reactive and is prone to free radical reactions. As an antioxidant, iodine can directly serve as an electron donor to quench reactive oxygen species (ROS). It can also be used as a free radical to iodinate tyrosine and unsaturated fatty acids on cell membranes, reducing their reaction with free radicals (40) to achieve the purpose of anti-oxidation. The reducing properties of iodine can help the production of thyroid hormones, maintain the normal function of the thyroid, and may also play a beneficial role in chronic metabolic diseases related to free radicals (41). Due to the interference of iodine nutrition, our research shows that in iodine-deficient populations, a correlation cannot be considered between TNs and MetS. Furthermore, in subgroups of different study types, MetS is positively correlated with TN; in subgroups of different diagnostic criteria, MetS is also positively correlated with TN.

Heterogeneity is inevitable due to the large number of cross-sectional studies included, so a random effects model was used to complete the analysis. When the Ayturk et al., Ding et al. and Moon et al. studies are removed, we can see that heterogeneity decreases from a high heterogeneity of 99% to a moderate heterogeneity of 65%. Although the heterogeneity is still present, it can also indicate that these studies are the source of most of the heterogeneity. A deeper reading of these study methodologies revealed that Ayturk et al. study restricted the region to iodine-deficient area, Ding et al. study restricted the population to rural people and Moon et al. study was a long-term multicenter study of up to six years. These differences in design may explain the substantial increase in heterogeneity. In addition, sensitivity analyses excluding each study separately showed that none of the studies affected the stability of this meta-analysis.

The main aim of this systematic review and meta-analysis was to elucidate the relationship between TNs and MetS through a rigorous statistical analysis. However, our systematic review and meta-analysis has some limitations. First, the number of relevant articles in this analysis was relatively small, and most of them were observational studies with a cross-sectional design, increasing the heterogeneity. Second, several relevant studies were not included in this systematic review and meta-analysis because they had incomplete original data. Third, our results are based on the data provided in the included articles, whereas a more accurate analysis could be performed if data on individual characteristics were available and adjustment could be performed for other covariates, such as smoking, alcohol consumption, and other lifestyle variables. In conclusion, the results of our meta-analysis suggest that MetS may be associated with an increased risk of TN that is independent of sex or age.

## Data Availability Statement

The original contributions presented in the study are included in the article/**Supplementary Material**. Further inquiries can be directed to the corresponding author.

## Author Contributions

CZ and XG: Conceived and designed the meta-analysis. CZ, XG, and YH: Searched and screened the relevant studies. CZ, XG, and YH: Estimated the quality of the studies. WT and ZS: Responsible for the nationwide cross-sectional study. CZ, XG, and YH: Responsible for the data analysis and manuscript writing. All authors contributed to the article and approved the submitted version.

## Funding

This work was supported by the National Natural Science Foundation of China (Grant No. 81970682).

## Conflict of Interest

The authors declare that the research was conducted in the absence of any commercial or financial relationships that could be construed as a potential conflict of interest.

## Publisher’s Note

All claims expressed in this article are solely those of the authors and do not necessarily represent those of their affiliated organizations, or those of the publisher, the editors and the reviewers. Any product that may be evaluated in this article, or claim that may be made by its manufacturer, is not guaranteed or endorsed by the publisher.
