# Supplementary material for: Correlation Between Thyroid Nodules and Metabolic Syndrome: A Systematic Review and Meta-Analysis
Source: Front Endocrinol (Lausanne). 2021 Sep 16;12:730279. doi: 10.3389/fendo.2021.730279 (PMC8481784; doi:10.3389/fendo.2021.730279)
Supplement: Supplementary file 1 [file Table_1.docx]

Supplementary Material

# Table S1. Results of quality assessment for the included cross-sectional studies

| Agency for Healthcare Research and Quality (AHRQ)  Item | Chen  2018 | | | Ding  2017 | | | Feng  2016 | | | Guo  2019 | | | Moon  2018 | | | Pan  2020 | | | Rendina  2012 | | | Su  2019 | | | Yin  2014 | | | Li  2019 | | | |
| --- | --- | --- | --- | --- | --- | --- | --- | --- | --- | --- | --- | --- | --- | --- | --- | --- | --- | --- | --- | --- | --- | --- | --- | --- | --- | --- | --- | --- | --- | --- | --- |
|  | Y | N | U | Y | N | U | Y | N | U | Y | N | U | Y | N | U | Y | N | U | Y | N | U | Y | N | U | Y | N | U | Y | N | U |  |
| 1) Define the source of information (survey, record review) | ★ |  |  | ★ |  |  | ★ |  |  | ★ |  |  | ★ |  |  | ★ |  |  | ★ |  |  | ★ |  |  | ★ |  |  | ★ |  |  |  |
| 2) List inclusion and exclusion criteria for exposed and unexposed subjects (cases and controls) or refer to previous publications | ★ |  |  | ★ |  |  | ★ |  |  | ★ |  |  | ★ |  |  | ★ |  |  | ★ |  |  | ★ |  |  | ★ |  |  | ★ |  |  |  |
| 3) Indicate time period used for identifying patients | ★ |  |  | ★ |  |  | ★ |  |  | ★ |  |  | ★ |  |  | ★ |  |  | ★ |  |  | ★ |  |  | ★ |  |  | ★ |  |  |  |
| 4) Indicate whether or not subjects were consecutive if not population-based | ★ |  |  | ★ |  |  | ★ |  |  | ★ |  |  | ★ |  |  | ★ |  |  | ★ |  |  | ★ |  |  | ★ |  |  | ★ |  |  |  |
| 5) Indicate if evaluators of subjective components of study were masked to other aspects of the status of the participants |  | ★ |  |  | ★ |  |  | ★ |  |  |  | ★ |  | ★ |  |  | ★ |  |  | ★ |  |  | ★ |  |  | ★ |  |  |  | ★ |  |
| 6) Describe any assessments undertaken for quality assurance purposes (e.g, test/retest of primary outcome measurements) | ★ |  |  |  | ★ |  | ★ |  |  |  | ★ |  | ★ |  |  |  | ★ |  | ★ |  |  | ★ |  |  | ★ |  |  |  |  | ★ |  |
| 7) Explain any patient exclusions from analysis | ★ |  |  | ★ |  |  | ★ |  |  | ★ |  |  | ★ |  |  | ★ |  |  | ★ |  |  | ★ |  |  | ★ |  |  | ★ |  |  |  |
| 8) Describe how confounding was assessed and/or controlled | ★ |  |  | ★ |  |  |  |  | ★ | ★ |  |  |  | ★ |  |  | ★ |  | ★ |  |  |  | ★ |  | ★ |  |  | ★ |  |  |  |
| 9) If applicable, explain how missing data were handled in the analysis |  | ★ |  |  | ★ |  |  | ★ |  |  | ★ |  |  | ★ |  |  | ★ |  |  | ★ |  |  | ★ |  |  | ★ |  |  | ★ |  |  |
| 10) Summarize patient response rates and completeness of data collection | ★ |  |  | ★ |  |  |  | ★ |  |  | ★ |  |  | ★ |  |  | ★ |  |  | ★ |  |  | ★ |  |  | ★ |  |  | ★ |  |  |
| 11) Clarify what follow-up, if any, was expected and the percentage of patients for which incomplete data or follow-up was obtained |  | ★ |  |  | ★ |  |  | ★ |  |  | ★ |  |  | ★ |  |  | ★ |  |  | ★ |  |  | ★ |  |  | ★ |  |  | ★ |  |  |
| ***Quality scores*** | 9 | | | 8 | | | 7 | | | 6 | | | 7 | | | 6 | | | 8 | | | 7 | | | 8 | | | 6 | | | |

Y, Yes; N, No; U, Unclear; an item would be scored ‘0’ if it was answered ‘NO’ or ‘UNCLEAR’; if it was answered ‘YES’, then the item scored ‘1’ (Question 5 take reverse scoring).

**
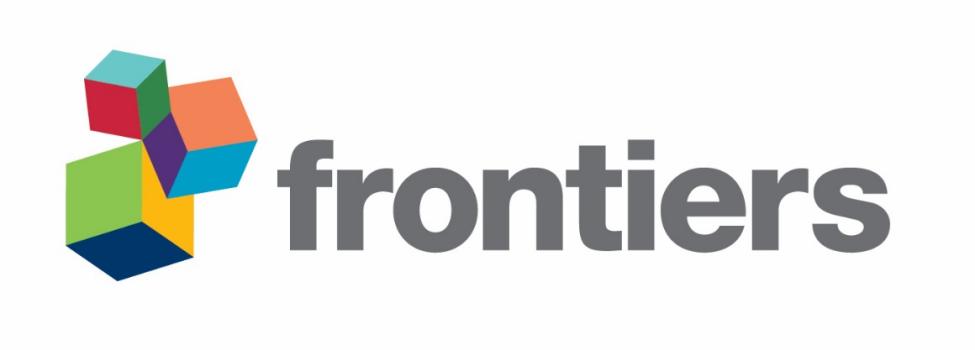
**
