# Supplementary material for: Correlation Between Thyroid Nodules and Metabolic Syndrome: A Systematic Review and Meta-Analysis
Source: Front Endocrinol (Lausanne). 2021 Sep 16;12:730279. doi: 10.3389/fendo.2021.730279 (PMC8481784; doi:10.3389/fendo.2021.730279)
Supplement: Supplementary file 2 [file Table_2.docx]

Supplementary Material

# Table S2. Results of quality assessment for the included case-control studies

|  |  | Ayturk  2009 | Liang  2020 | Shin  2016 |
| --- | --- | --- | --- | --- |
| ***Selection*** | Adequate definition of cases | ★ | ★ | ★ |
|  | Representativeness of the cases | ★ | ★ | ★ |
|  | Selection of controls | ★ | ★ | ★ |
|  | Definition of controls | ★ | ★ | ★ |
| ***Comparability Control for important factors*** | | ★★ | ★★ | ★★ |
| ***Exposure*** | Ascertainment of exposure | ★ | ★ | ★ |
|  | Same method of ascertainment for cases and controls | ★ | ★ | ★ |
|  | Non-response rate | - | - | - |
| ***Quality scores*** | | 8 | 8 | 8 |

**
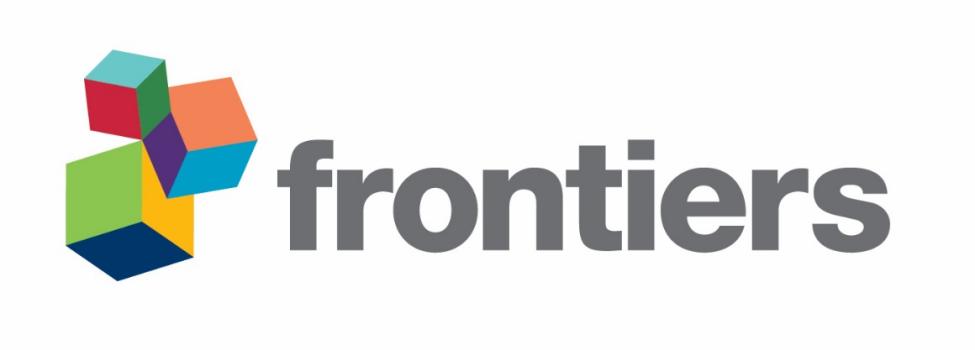
**
